# Peatland groundwater level in the Indonesian maritime continent as an alert for El Niño and moderate positive Indian Ocean dipole events

**DOI:** 10.1038/s41598-023-27393-x

**Published:** 2023-01-18

**Authors:** Albertus Sulaiman, Mitsuru Osaki, Hidenori Takahashi, Manabu D. Yamanaka, Raden Dwi Susanto, Sawahiko Shimada, Keiji Kimura, Takashi Hirano, Rahmawati Ihsani Wetadewi, Silsigia Sisva, Tsuyoshi Kato, Osamu Kozan, Hideyuki Kubo, Awaluddin Awaluddin, Nobuyuki Tsuji

**Affiliations:** 1 Research Center for Climate and Atmosphere, National Research and Innovation Agency, Bandung, Indonesia; 2 Research Faculty of Agriculture, Hokkaido University, Sapporo, Japan; 3 Hokkaido Institute of Hydro-Climate, Sapporo, Japan; 4 Research Institute for Humanity and Nature, Kyoto, Japan; 5 Department of Atmospheric and Oceanic Science, University of Maryland, College Park, MD 20742 USA; 6 Faculty of Fisheries and Marine Science, Diponegoro University, Semarang, Indonesia; 7 Faculty of Regional Environment Science, Tokyo University of Agriculture, Tokyo, Japan; 8 Department of Geography, Nara University, Nara, Japan; 9 Peatland and Mangrove Restoration Agency, Jakarta, 10350 Indonesia; 10 PT Wana Subur Lestari/PT Mayangkara Tanaman Industri, Jakarta, 10270 Indonesia; 11 Sumitomo Forestry Co. Ltd., Tokyo, Japan; 12 Center for Southeast Asian Studies, Kyoto University, Kyoto, Japan; 13 Institute for Global Environmental Strategies (IGES), Kanagawa, Japan; 14 NPO FutureForest Institute, Yanagawa, Japan

**Keywords:** Climate change, Ocean sciences, Physical oceanography, Atmospheric science, Atmospheric dynamics

## Abstract

In general, it is known that extreme climatic conditions such as El Niño and positive Indian Ocean Dipole (IOD+) cause prolonged drought in Indonesia's tropical peatlands so that groundwater levels (GWL) drop and peat is prone to fire. However, 27 years of GWL measurements in Central Kalimantan peat forests show the opposite condition, where the lowest GWL occurs several weeks before El Niño and after IOD+ reaches its peaks. We show that the dropped sea surface temperature anomaly induced by anomalously easterly winds along the southern Java-Sumatra occurs several weeks before the GWL drop to the lowest value. Local rainfall decreased, and GWL dropped sharply by 1.0 to 1.5 m, during the super El Niño events in 1997/98 and 2015, as well as remarkable events of IOD+ in 2019. It is suggested that the tropical peatland ecohydrological system (represented by the GWL), El Niño Southern Oscillation (ENSO), and IOD+ are teleconnected. Hence, monitoring GWL variability of peatland over the IMC is a possibility an alert for extreme climate events associated with El Niño and/or moderate IOD+.

## Introduction

**Figure 1:**
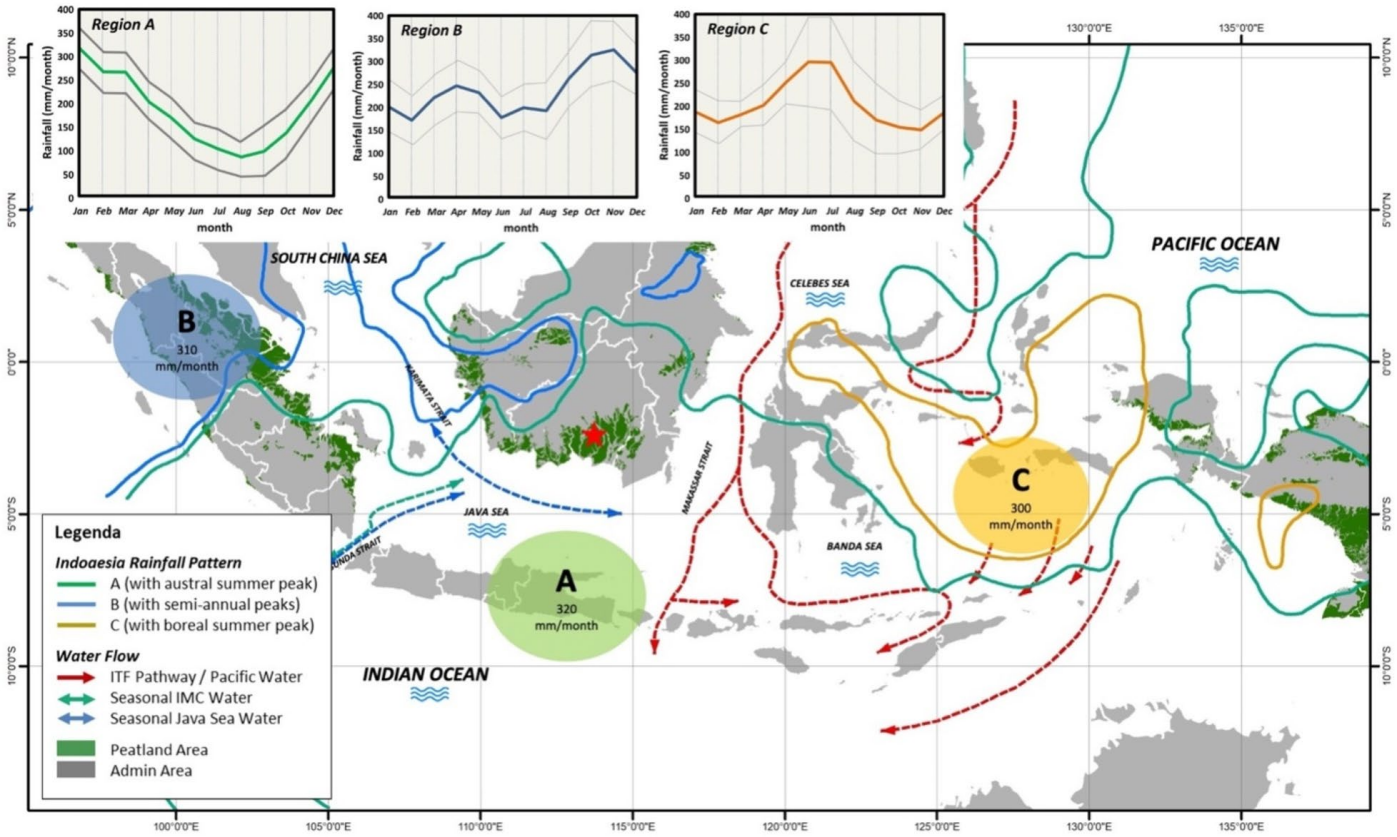
Map of the Indonesian Maritime Continent (IMC) and peatland distribution in Indonesia generated using QGIS 3.26.1(https://www.qgis.org/en/site/forusers/download.html). The map is overlaid with three (A, B, C) annual rainfall patterns with the amount of mean annual rainfall^1^ (with modified legend and written permission from the publisher, John Wiley and Sons) and the Indonesian Throughflow (ITF) pathways (red, blue and green arrows)^2^. The red star in south Kalimantan denotes the location of GWL station.

The Indonesian Maritime Continent (IMC), many large and small islands surrounded by the world’s warmest seawater, plays an essential role in the global climate. With its complex geography and narrow passages, IMC provides the only pathway for low-latitude Pacific Ocean water to flow into the Indian Ocean. Transport and water-mass transformation associated with the Indonesian Through Flow (ITF) directly impacts heat and freshwater budgets of the Pacific and Indian Oceans and may have feedback on Asian-Australian monsoons, El Niño Southern Oscillation (ENSO), and Indian Ocean Dipole (IOD) (Fig. 1^1–8^). The active convection over the IMC drives the Walker circulation, and hence sea surface temperature anomaly (SSTA) in this region impacts global weather and climate^9–13^. Consequently, the land/islands of Indonesia that straddle along the equatorial band and air–sea interactions over the IMC have a direct link to global ocean circulation and climate via atmospheric and oceanic teleconnections. Intensive solar radiation and abundant water cause IMC to play an essential role in global equatorial circulation. Within the Earth's climate system and the water cycle, latent heat is inputted from the ground to the atmosphere through the cloud-precipitation process, connecting the land to the oceans through the atmosphere^14^. In the equatorial region with the meridional rainfall peak of approximately 2000 mm/year^15^, the IMC produces most active convective clouds and rainfall on Earth (approximately 2700 mm/year)^16,17^ followed by Central America. Since the most dominant mechanism of convective cloud generation is the diurnal cycle induced by land-sea temperature contrast, any local land modification, as well as SSTA due to El Niño^18,19^, changes rainfall of the IMC's tropical peatlands with large water reservoirs, and finally changes the global circulation and climate^14,19^.

The three largest islands of the IMC (Borneo/Kalimantan, Papua, and Sumatra) have peatlands (Fig. 1), an important ecosystem in the world climate. Figure 1 also shows three annual rainfall patterns with the IMC^1^ and the ITF pathways^2^. Peatlands currently cover 3% of the global land surface and have an essential impact on global climate change through carbon sources in this century^20–23^. Land-use change in the last decade has played a significant role in triggering high greenhouse gas (GHG) emissions. It was estimated that by 2100 there would be an additional 249 ± 38 Pg (1 Pg = 10^15^ g) emissions due to changes in peatlands^22^. On the other hand, the lowering of groundwater level (GWL) driven by a warming climate and anthropogenic forces increase greenhouse gases by 0.86 (0.36–1.36) Pg CO_2_-equivalent year^−1^ at the end of the twenty-first century^24,25^. However, the role of tropical peat on the global carbon cycle and its possible linkage to climate is still not fully understood due to limited measurements.

Climate extremes such as El Niño and positive Indian Ocean Dipole (IOD+) significantly impact the tropical peat forest ecosystem in IMC^17–19^, especially on the IMC's tropical peatlands because their ecosystems have large water reservoirs^20^. The connection between the IMC and the global circulation means that this circulation impacts the peatland in the IMC. When El Niño occurs, most peatlands experience severe drought, decreasing the GWL^22^. Lowering the GWL leads to a linear increase in soil respiration, especially in drained tropical peatlands, and increased CO_2_ emissions^26,27^. It was observed that CO_2_ emissions increased by 79 to 238 gC m^−2^ per 0.1 m decrease in the GWL^28–30^. Based on statistical modeling, it has been shown that the fire strength will increase by considering hydrological conditions, especially during strong El Niño events^32^. Hot spot and precipitation data with a space-borne Moderate Resolution Imaging Spectroradiometer (MODIS for ten years (2002–2011) shows that peat fires occur in El Niño conditions and reach their peak in the driest conditions (September/ October in those years) in Kalimantan^33^. While in Central Kalimantan, data on precipitation, hotspots, and GWL (obtained from the model) for the period 1997–2007 show that most hotspots occur when the GWL drops by more than 0.4 m. The data also indicates that precipitation reaches a minimum of about 2 months before GWL reaches its lowest condition^34^. Recently, Deshmunk et al. (2021) measured carbon dioxide and methane emissions between 2017 to 2020 and showed an increase in emissions from peatlands due to extreme drought caused by the positive IOD phase in conjunction with El Niño^28^. Those previous studies on the IMC peatland were carried out in a limited period, such as the drought, fires, and carbon emissions from peat decomposition caused by a specific climate^26–28,30,31,33,34^. However, if the temperature and moisture of local land are modified irreversibly by the peatland fire, the cloud generation over IMC, and finally, the global climate should also be modified irreversibly.

In this paper, we compare our long-term measurements of GWL and rainfall in the natural tropical peatlands in Kalimantan with SSTA and winds associated with extreme climate events such as El Niño and positive IOD. Peatland modification indeed triggers irreversible climate changes. We hypothesize that tropical peatland ecosystems (represented by the GWL) and ENSO are teleconnected, although the corresponding teleconnection mechanisms are still not fully resolved. This paper is the first report to show evidence of GWL decreasing before the El Niño and moderate IOD+. We believe this should be confirmed and explained by more observations and theories. In this paper, we describe that this is possible, based on many recent studies showing that local hydro-meteorological processes govern the convective activity on the land of IMC and affect essentially broader climates concerned with ocean–atmosphere interaction, including ENSO and IOD+.

## Methods

To answer the hypothesis described in the previous section, we installed a special station to carry out long-term monitoring of GWL and rainfall in the peatland of Indonesia. We compared those data with regional weather/climate (monsoon, ENSO, and IOD), SSTA, ITF, and wind-induced upwelling variability, which should influence the GWL variability.

**Figure 2:**
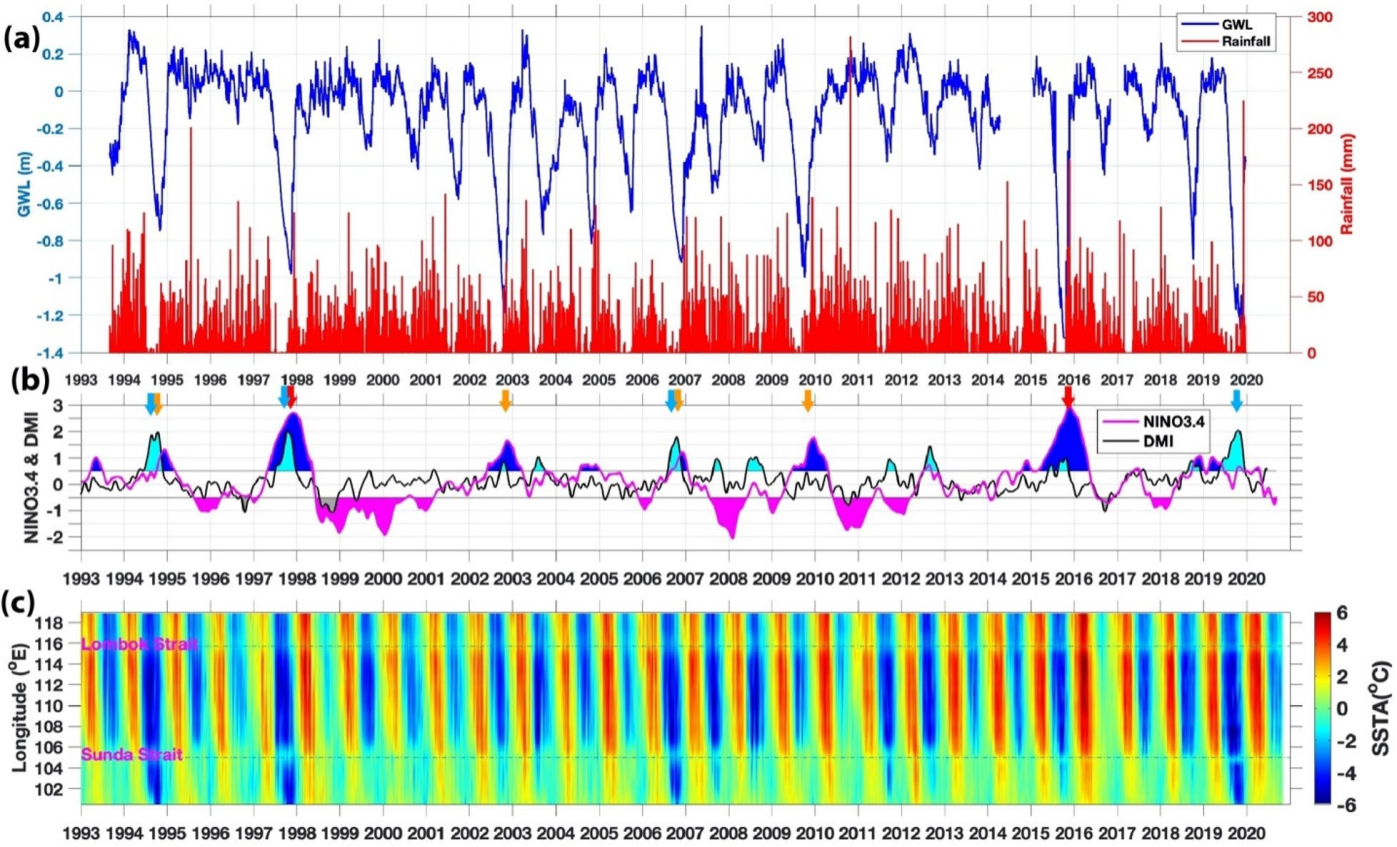
(**a**) Time series of the groundwater level (GWL) and rainfall in Central Kalimantan. (**b**) Nino3.4 and Dipole Mode (DMI) indices. The shaded area above and below the 0.5 values for Nino3.4 (blue & magenta), and DMI (cyan and gray). Brown arrows (El Niño events in 1994, 1997, 2002, 2006, 2009); Red arrows (Super El Niño events in 1997 & 2015). Blue arrows (IOD+ events in 1994, 1997, 2006, 2019); (**c**) the sea surface temperature anomaly (SSTA) along the southern coast of Sumatra-Java. Super El Niño, when Nino3.4 above +2.5, occurred in 1997 and 2015.

The special station of GWL and rainfall data are located at the peatland of *Taman Nasional Sebangau* (Sebangau National Park) near Palangkaraya, Central Kalimantan Province (2.321002°S, 113.901161°E) (Fig. 2). The daily GWL was measured by a pressure sensor from September 1, 1993, until December 12, 2019^35^. The site vegetation cover includes evergreen overstory trees, shrubs, and young trees. The site is a tropical ombrotrophic bog peatland composed mainly of roots and remains of trees, and the average peat depth is greater than 4 m. Precipitation is the primary source of water in the peatland. The large trees (> 2 m diameter) in the national park were logged during the early 1990s, and shallow trenches were prepared to carry the logged trees. The area was protected after the late 1990s, and the shallow ditches were quickly buried naturally.

The ENSO phase time series is based on the Niño3.4 index, an average SSTA over the central/eastern equatorial Pacific (5^o^ S–5^o^ N, 170–120^o^ W), and available from NOAA (https://www.cpc.ncep.noaa.gov/data/indices/). The strength of ENSO is divided into four: normal, weak, strong, and extreme when Niño3.4 is less than ± 0.5 °C, between ± [0.5–1] ^o^C, ± [1.0–1.5] ^o^C, larger than ± 1.5 °C (https://ggweather.com/enso/oni.htm). Super El Niño is defined when Niño3.4 above 2.5 °C, occurred in 1972, 1982, 1997, and 2015^40^. The Dipole Mode Index (DMI) is the mean SSTA difference between the western (10^o^ S–10^o^ N, and 50–70^o^ E) and eastern (10–0^o^ S, 90–110^o^ E) Indian Ocean^5^, available at https://stateoftheocean.osmc.noaa.gov/sur/ind/dmi.php. Most of the time (though not always), El Niño events coincide with positive IOD, while La Niña events coincide with negative IOD. The impacts of ENSO on precipitation vary over the IMC region^14^. El Niño/IOD + causes much less rainfall and increases the likelihood of forest fire over the IMC. On the other hand, the La Niña/IOD^–^ causes much more rain and raises the possibility of flooding and overflow on the GWL measurements. Hence, we concentrate on the El Niño/IOD+ events in the following sections.

**Figure 3:**
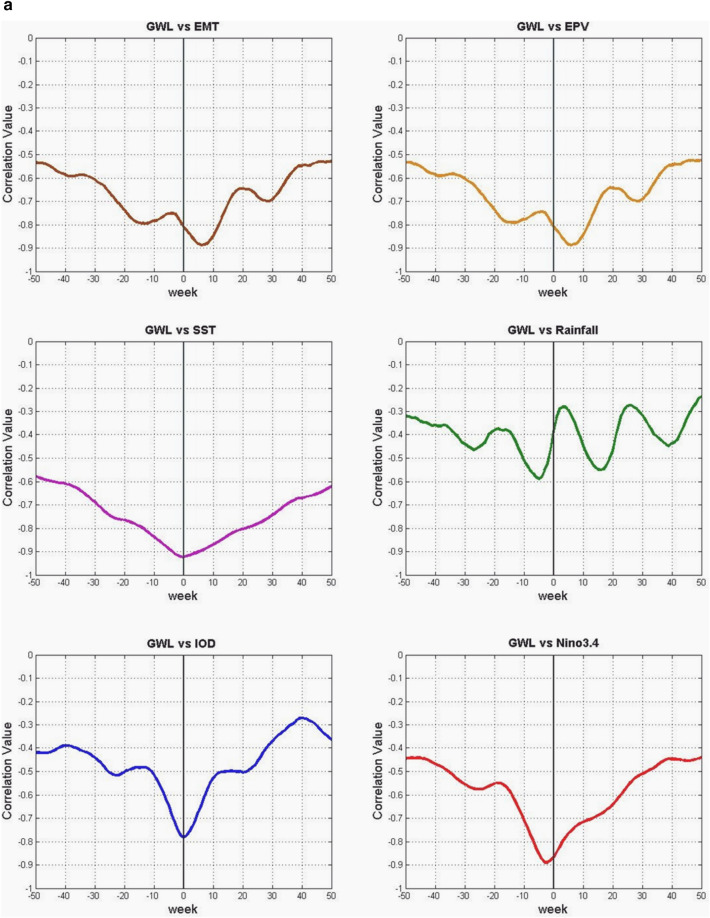

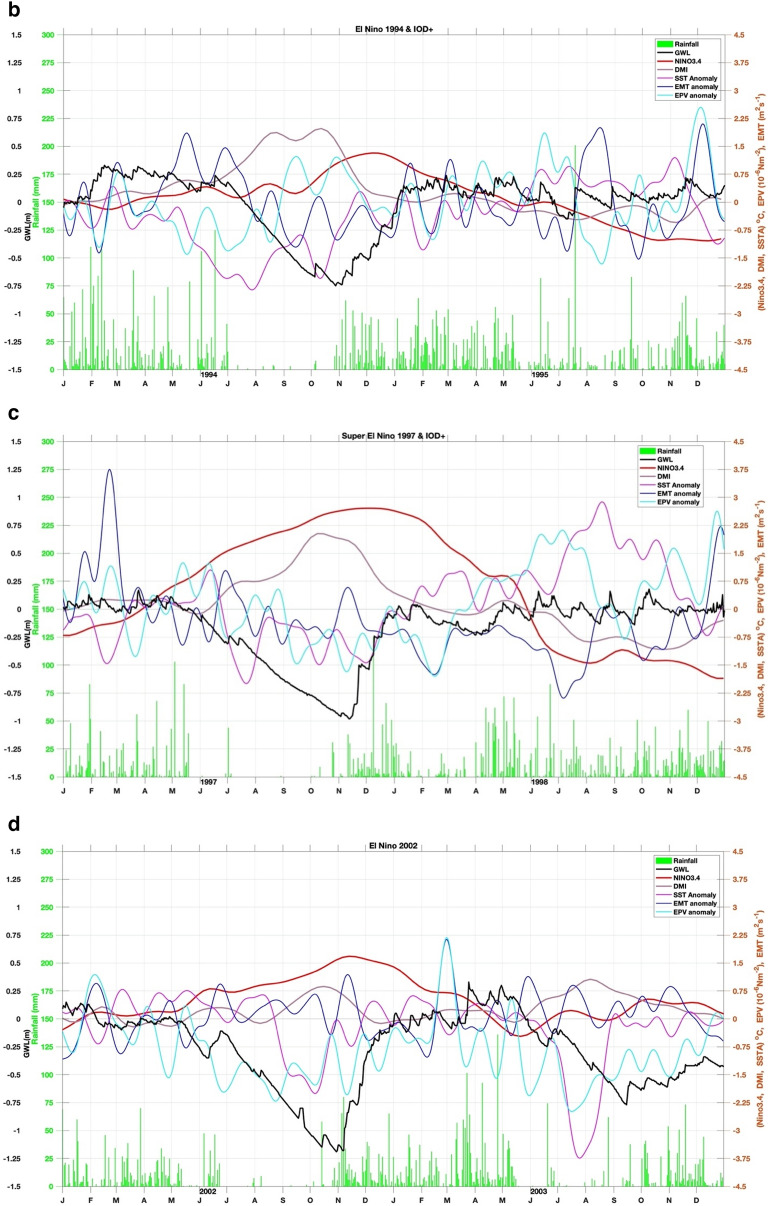

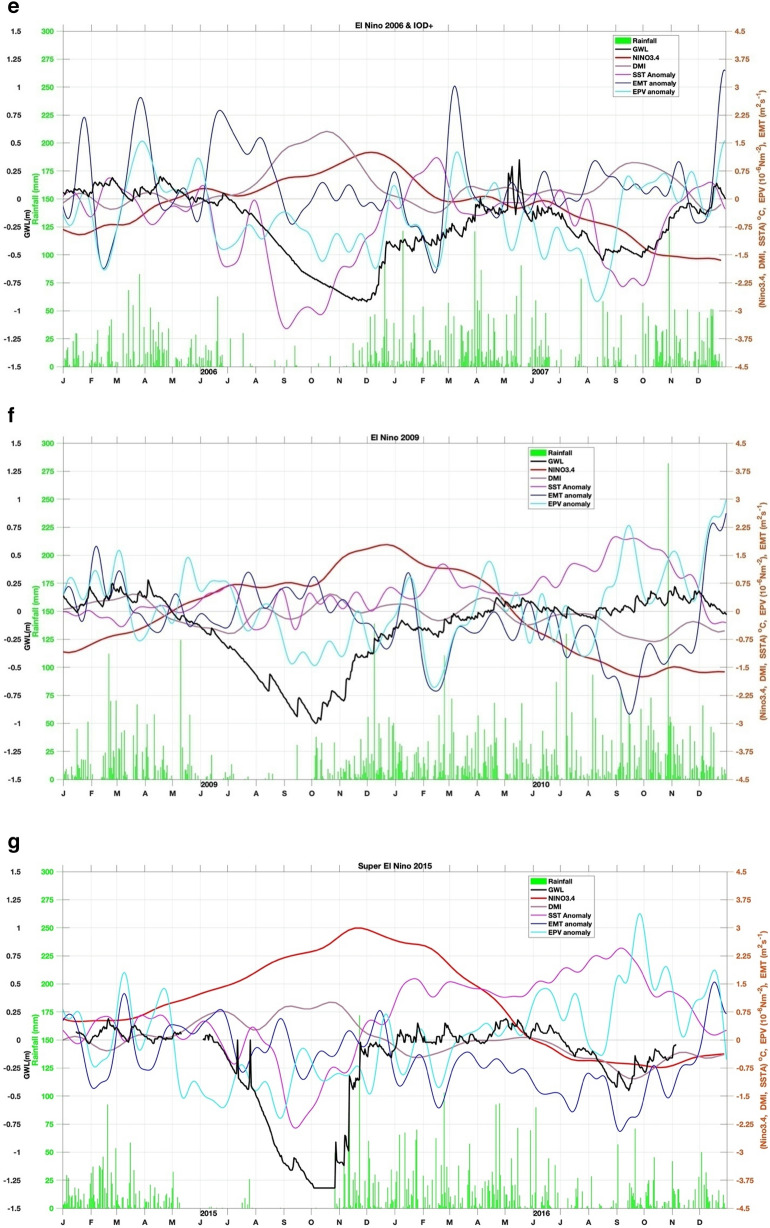

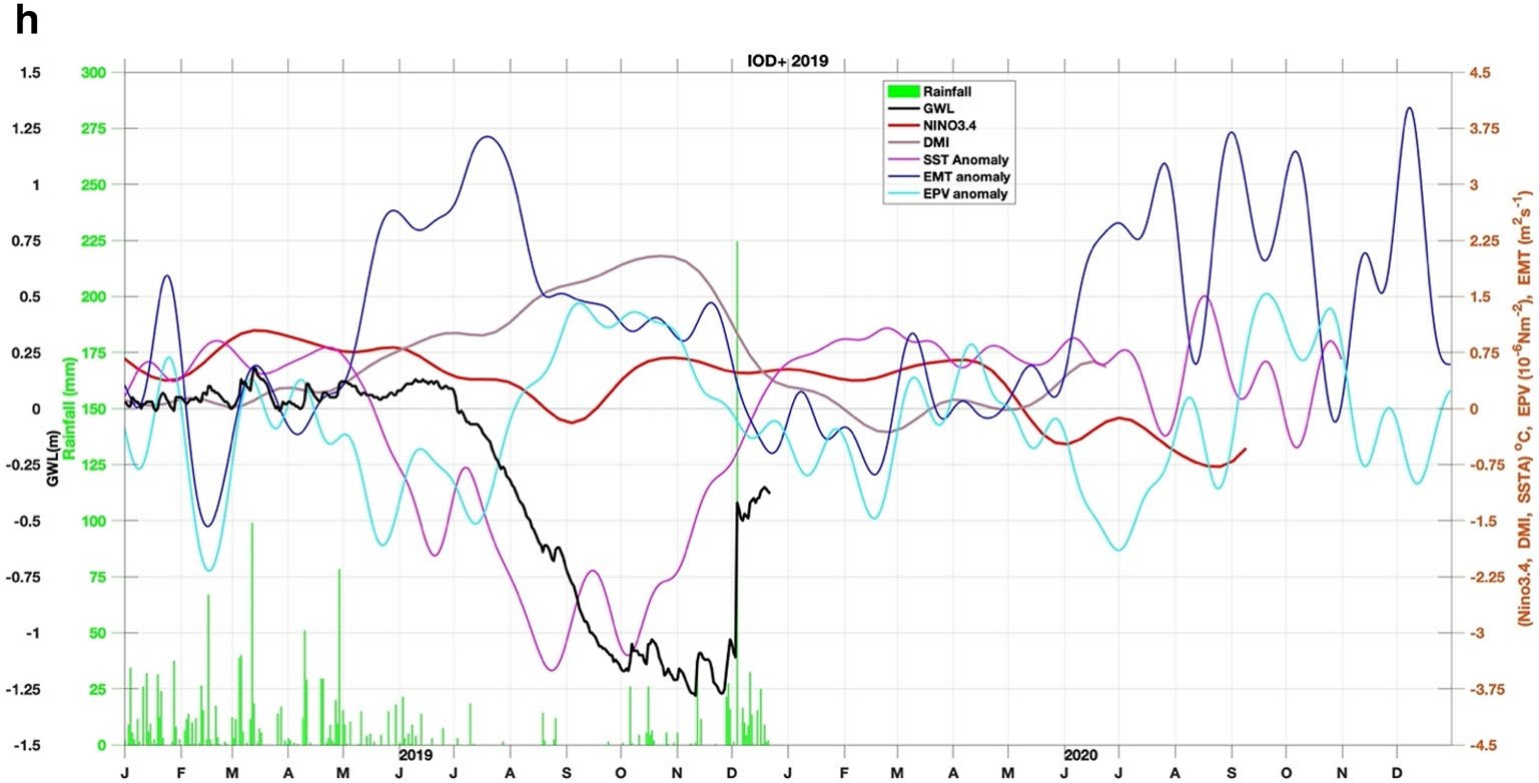
(**a**) Correlation between GWL and EMT, EPV, SST, Rainfall, DMI and Nino34, during El Niño and IOD events. (**b**) Time series of GWL (black line), rainfall (green bar), Nino34 (red), DMI (purple), SST (magenta), Ekman mean transport (EMT) (blue), and Ekman pumping velocity (EPV) (cyan) anomalies for 1994 weak El Niño but strong Indian Ocean dipole positive (IOD+) event. (**c**) Similar to (**b**) but for the 1997 Super El Niño event. (**d**) Similar to (**b**) but for the 2002 El Niño event. (**e**) Similar to (**b**) but for the 2006 El Niño and IOD+ event. (**f**) Similar to (**b**) but for the 2009 El Niño event. (**g**) Similar to (**b**) but for the Super 2015 El Niño event. (**h**) Similar to (**b**) but for the 2019 IOD+ event.

Moreover, we also calculate SSTA south of Java-Sumatra, where seasonal monsoon winds-induced upwelling occurs (Fig. 2; i.e.^36,37^). During the southeast monsoon (boreal summer), easterly wind- induced upwelling. Meanwhile, during the boreal winter, winds-induced downwelling. IOD and ENSO modulate this upwelling. Anomalously stronger early wind during IOD positive and El Niño enhance the upwelling strength and expand the upwelling up to western Sumatra (Fig. 2^36^). To quantify the wind- induced upwelling (Fig. 3), we calculated the variability of Ekman dynamics which consist of Ekman Mass Transport (EMT) and Ekman Pumping Velocity (EPV) based on wind stress and wind curl along the southern coast of Java, respectively^38,39^. Climatological means of EMT and EPV were removed to obtain their anomalies.

## Results

The average GWL for nearly three decades is –0.12 m (see Fig. 2). The rainfall and GWL decrease in the dry season (typically June–October) in Central Kalimantan but abruptly reduced in 1997, 2002, 2006, 2009, 2015, and 2019 and moderately in 1994, 2003, 2004, 2005, and 2018. When the GWL decreased either abruptly or moderately (larger than –0.7 m), during El Niño and/or moderate IOD+ events. The average GWL minima before 2010 (–0.9 to –1.0 m) and deepen after 2015 (–1.3to –1.4 m). An extreme decrease in rainfall always precedes every severe GWL decrease, but the differences between before 2010 and after 2015 are unclear. Thus, the GWL deepening is more significant than the rainfall decrease. As hypothesized in the Introduction, the first trigger of IMC dryness is prolonged anomalous easterly winds that induce upwelling and colder SSTA along the southern Java-Sumatra coastline, as shown in the lower panel of Fig. 2. The local diurnal cycle (mainly land breeze/mountain wind in the evening, which is also easterly along the western coasts of each major island) may contribute to the intensification of the sea-surface wind and, thus, the ocean upwelling. During moderate or extreme IOD+ events (1994; 1997; 2006; 2019) cooler SST appeared from the south of Java to the west of Sumatra. When the GWL decreased sharply, the IMC area's atmosphere was dry, and the SSTA (especially south of Java) generally cooled before the GWL. The GWL dropped significantly after super El Niño 2015^40^ and during extreme IOD+ event in 2019. 

The correlations between GWL and the climate parameters [Ekman Mass Transport (EMT), Ekman Pumping Velocity (EPV), SST, rainfall anomaly, Niño 3.4, and DMI] are depicted in Fig. 3a. The correlation between the GWL and Nino3.4 is 0.89, in which the GWL leads Nino3.4 by 3 weeks. Meanwhile the GWL dropped almost at the same time as the DMI. The correlation between the GWL and SST is 0.91, in which SST the same time with GWL dropped. The correlation between the GWL and rainfall is 0.58. Meanwhile the correlation between the GWL and winds (represented by EMT and EPV) is 0.88 and 0.87, with winds lead the GWL by 7 weeks. All correlations values are above the 99% confidence level. The seasonal reversal of monsoon winds is significantly observed over the IMC, i.e., the north-westerlies during December–February and the strong south-easterly during June–August and prolonged to October during the El Niño and IOD+^14,36,41^. The maximum values of negative wind stress curl or upward EPV are observed during June–October, which leads to enhanced upwelling and cooling of the SST (negative SSTA). These results support our hypothesis that tropical peatland ecosystems (represented by the GWL) and ENSO are teleconnected. Therefore, monitoring GWL is essential for understanding the onset of ENSO and IOD.

Detailed relations between GWL and EMT Anomaly (EMTA), EPV Anomaly (EPVA), SST Anomaly (SSTA), rainfall, Niño 3.4, and DMI, during El Nino and/or moderate IOD+ events are presented in Fig. 3b–h. In the event of El Niño 1994 (see Fig. 3a,b), EMTA reached its maximum in May, followed by a decrease in SST in August. EPVA did not show significant variation. GWL reaches its lowest depth (–75 cm calculated from the peat surface) in November, while El Niño peaks in mid-December and DMI in October. This year the dry season occurs from July to November. In the 1997 El Niño event (see Fig. 3b,c), the EMTA peaked in March, while the minimum SSTA occurred in mid-July. GWL reached its lowest condition (–1 m) in mid-November, while the peak of El Niño occurred in mid-December.

On the other hand, DMI peaked in October, and EPVA did not show significant variation. This year the dry season occurs from mid-May to mid-November. In the 2002 El Niño event (see Fig. 3c,d), GWL fell significantly –1.2 m in November, and El Niño peaked in mid-November. The decline in SSTA in October while EMTA and DMI did not show a significant pattern unless EPVA peaked around March of the following year. This year the dry season occurs from July to early November. During the 2006 El Niño event (Fig. 3d,e), EMTA peaked in April, SSTA dropped to its lowest in September, while GWL reached its minimum (–0.8 m) in late November. El Niño peaks in early December with a maximum DMI in October with an amplitude greater than Nino34. This year the dry season occurs from July to mid-November. The 2009 El Niño event (Fig. 3e,f) showed no significant variation in DMI, EMT, SST, and EPV anomalies. Still, the minimum GWL drop (–1 m) occurred in early October, with Nino34 reaching its peak in late December. This year the dry season occurs from July to October. In the 2015 El Niño event (Fig. 3f,g), EMTA and EPVA peaked in March, followed by a decline in SST in September. GWL reached its lowest condition (–1.35 m) at the end of October, whereas Nino34 reached its maximum at the end of November. There is no significant variation in DMI, and the dry season occurs from May to November. The event in 2019 (Fig. 3g,h) was not an El Niño year, but the DMI was relatively high at more than 2 °C, and peaked in early November when GWL fell to its lowest condition (–1.25 m) at the end of December. This year the peak of EMT occurred in July, and SST fell in September. This year dry season occurs from July to October.

In general, GWL will drop several weeks before Nino34 reaches its maximum in El Niño events. The above conditions enormously occur during super El Niño, namely Nino34 is greater than or equal to 2.5^o^C^40^. The EMTA parameter also rose several months earlier, and the SSTA in southern Java fell several months before the GWL reached its minimum. The SSTA pattern follows the GWL pattern with little variability from all events, less than 1 °C, while DMI and Niño3.4 were the opposite. In the 1997 El Niño event, the DMI peak rose four weeks before GWL dropped to the lowest depth of –0.95 m, while a peak of Niño3.4 occurred three weeks later. In the 2002 El Niño event, the DMI peak occurred almost simultaneously with the lowest GWL of –1.16 m, and the Niño3.4 peak occurred four weeks after the lowest GWL. In the 2006 El Niño event, the DMI peaked three weeks before the lowest GWL reached –0.9 m depth, and two weeks later, Niño3.4 peaked. Different conditions occurred in El Niño in 2009, where DMI peaked four weeks after GWL reached its lowest depth of –0.98 m. Meanwhile, the peak of Niño3.4 occurred more than two months from the lowest GWL. However, during the 2015 super El Niño, the DMI peak reappeared four weeks before the lowest GWL with a depth of –1.32 m, and the peak of Niño3.4 appeared five weeks later. An interesting thing happened in 2019 when the GWL reached a depth of –1.24 m which was not an El Niño year, but the DMI had a high amplitude of 2.2 °C (extreme IOD), which occurred three weeks before the lowest GWL. If GWL changes before a climate event, we should consider that the peatland modification indeed triggers irreversible climate change. 

## Discussion

Using in-situ measurement data over 27 years, we find that the GWL falls before El Niño reaches its peak. Studies of the interconnection of peat with global warming have begun to be carried out by many researchers because peatlands store a third of terrestrial organic carbon. Rafat et al*.* (2021)^20^ show that peatland carbon loss has a positive climate feedback loop based on 13 years of continuous eddy covariance flux measurements from Mer Blue Bog, Canada. Using a radiative forcing model and areal data from the Global Peatlands Database shows that forcing CH_4_ emission due to rewetting activities does not damage climate change. On the other hand, delaying wetting will increase the long-term global warming effect through sustainable CO_2_ emissions^22^.

In this paper, we use time series data to explain the teleconnection mechanism. The emergence of EMT during the southeast monsoon causes a mass of cold water to the surface so that SST will be cold, which causes reduced evaporation^38^. The reduced water vapor will cause the central part of Indonesia (including Kalimantan) to experience a decrease in rainfall which causes GWL to drop. On the other hand, the presence of EPV, with the combination of La Niña and the IOD negative events, tends to attenuate coastal EMT and EPV, but EMT and EPV tend to be strong during El Niño and positive IOD. In addition to CO_2_ emissions, the role of peatlands in the world's climate is through climate parameters such as evapotranspiration. A study using the observation of 95 eddy covariance towers showed that peatlands' evapotranspiration (ET) increased more than the ET of forests with increasing vapor pressure deficit (VPD). At high VPD over 2 kPa, the ET of peatlands exceeds the ET of the forest by up to 30%. Therefore, the peatland ET should be included in Earth system models to avoid biases in water cycle projections^23^. In this paper, we propose the teleconnection between tropical peatland and El Niño, of which the key processes are as follows:

- The study area is strongly influenced by the monsoon system, where the wind changes twice a year, namely, easterly wind (June–July–August (JJA)) and westerly wind (November–December–January). Anomalous easterly winds become prolonged and intensify during the austral winter (JJA, or boreal summer) until November or December.
- Anomalous easterly winds carry drier (low-humidity) air induced upwelling along the southern coast of Java and extent to west Sumatra and cool the SST region. Hence, it enhances positive feedback of SST (cooler SST causes low humidity)^36^, low rainfall, and a drastic decrease in the GWL. Therefore, negative SST anomalies occur primarily before the GWL troughs.
- Atmospheric convection moves eastward toward the middle-eastern tropical Pacific, and westerly wind bursts intensify. Simultaneously, oceanic Kelvin waves generated in the western Pacific propagate and carry warm water from the western Pacific warm pool eastward.

**Figure 4:**
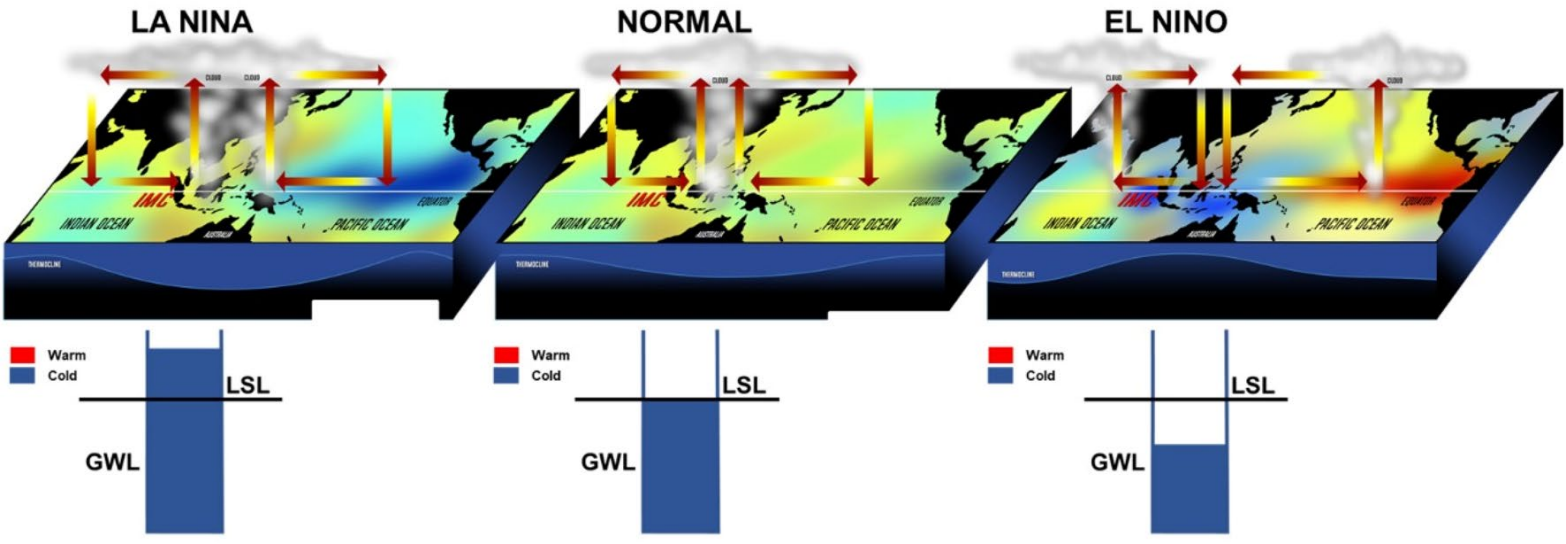
Diagram of the interconnections among energy, the water cycle, and SSTA over the IMC, Pacific Ocean, and the Indian Ocean along the equator during ENSO and IOD events. GWL is groundwater level, and LSL is land-surface level. The GWL dropped a few weeks before the El Nino event.

Consequently, the dry IMC area, including where the GWL decreased in the studied peatland, would affect El Niño with a time delay of a few weeks, and the IOD peak occurs a few weeks before the lowest GWL. A model of the IMC interconnections with El Niño and IOD events is proposed (Fig. 4). In the case of Normal and La Nina events, evaporations over the IMC from the sea are high, and this moisture is transported to the coastal zone and near-coastal inland region where heavy precipitation occurs. As a result of the subsequent high plant growth, high evapotranspiration rates occur, causing water to circulate in the coastal zone and inland. Such an extensive water reservoir, vigorous plant growth, and restricted litter decomposition cause tropical peatlands to form large carbon reservoirs. IMC has a unique role both locally and internationally global weather climate system. Inaccuracies in future weather and climate predictions are due to our lack of understanding of the critical processes that govern this role, so this happens to bias and systematic error in constructing a numerical model for the region^42,43^. Research shows a strong indication that extreme ENSO events may become more frequent in the future when external forcing increases (greenhouse gases, aerosols, solar variability)^43–45^.

**Figure 5:**
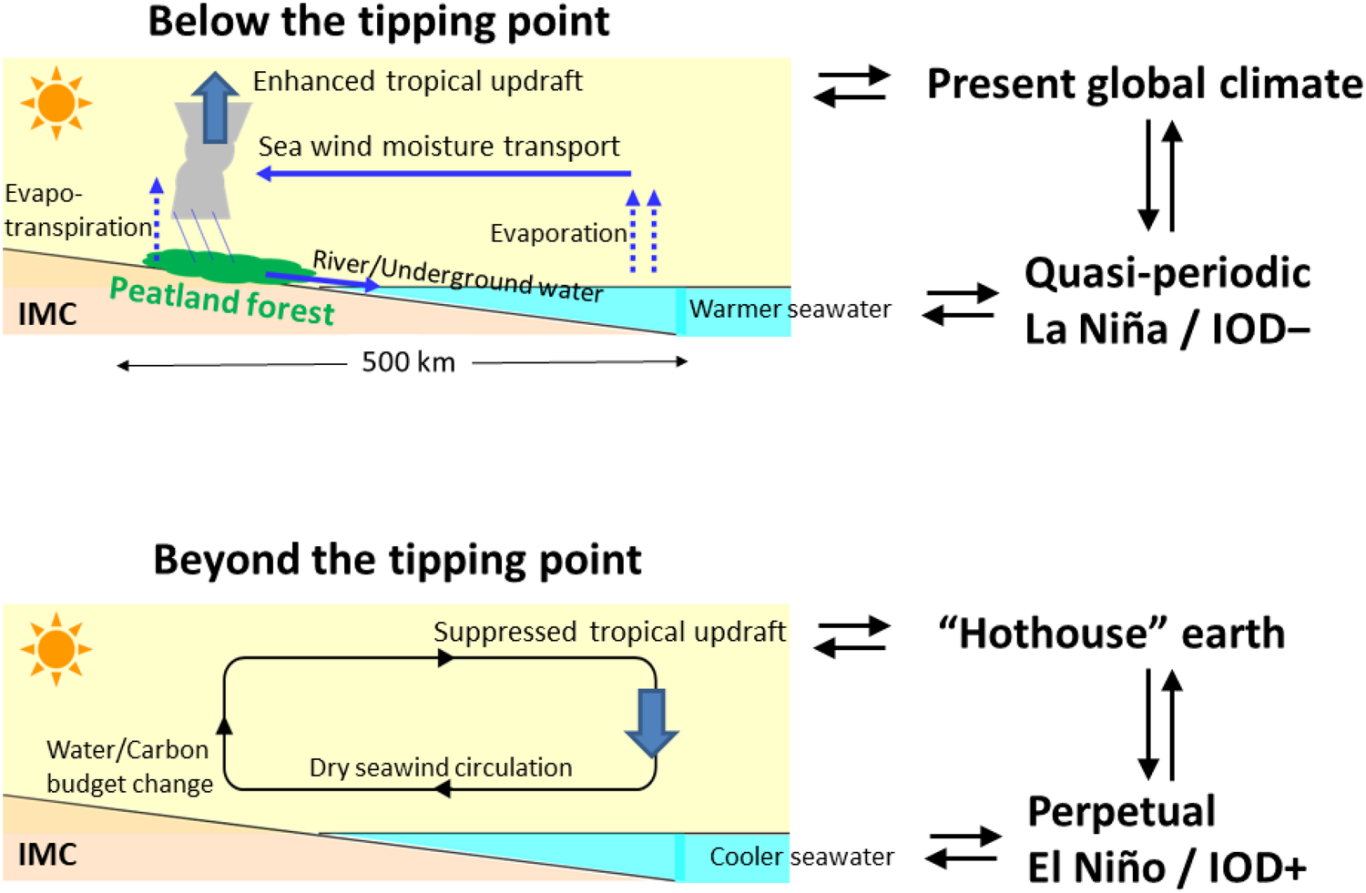
Diagram of the mechanisms of the IMC peatland—global climate link through the sea–air–land interactions. This response to two re-acknowledged processes between IMC convection—global atmospheric circulation and between IMC land water—tropical ocean, in addition to the well-studied process between tropical ocean—global atmosphere (TOGA). (**a**) Condition for the present climate with the IMC peatland forest and wetness (high GWL), convection and rainfall are generated by the land–sea temperature contrast (with diurnal cycle) and sufficiently wetland surface (high humidity in the lower atmosphere) in the IMC. The sea–surface temperature varying with ENSO and IOD makes flood (no-fire) and drought (peat and fire) well known. (**b**) Condition if the peatland is more and more degraded, the dry land suppresses convection in the IMC. Such suppression of the IMC convection appears in the El Niño (including Modoki) or IOD+ phases naturally at present, but the peat land degradation by human activities causes it perpetually, which makes the global climate (predicted as hothouse) beyond the tipping point.

Finally, we propose a hypothesis that GWL in tropical peat may be used as a parameter to determine the tipping point of anthropogenic effects on climate change in the IMC (see Fig. 5). The modification of peatland changes the local hydro-meteorological processes (as immediately indicated by GWL, and followed by modified diurnal-cycle sea-land breeze circulation^14,17^), which changes the total convective activity on land of IMC. When peat forests exist with high GWL, evaporation is intensive so that many convection clouds are formed, strengthening the tropical updraft, which will be involved in global circulation. The condition is the opposite if GWL decreases where the intensity of evaporation decreases, resulting in a weakening of the tropical updraft, which will impact global circulation. By studying this behavior intensively, we can finally determine a tipping point of the planetary boundary proposed by Steffen et al.^46^.

## Data Availability

The sea surface temperature (SST) data is publicly available from NASA-PODAAC drive https://podaac.jpl.nasa.gov/dataaccess. The wind data is publicly available from the Copernicus Marine Service, https://resources.marine.copernicus.eu/products. The Nino index is publicly available from the NOAA Climate Prediction Center (https://www.cpc.ncep.noaa.gov/data/indices/), while the Dipole Mode Index is publicly available from https://stateoftheocean.osmc.noaa.gov/sur/ind/dmi.php. The monthly in situ groundwater level and rainfall data sets will be publicly available two years after the completion of the collaborative “Japan-Indonesia Collaboration Research of Tropical Peatland” project through the National Research and Innovation Agency (BRIN).

## References

[1] Aldrian E, Susanto RD (2003). Identification of three dominant rainfall regions within Indonesia and their relationship to sea surface temperature. Int. J. Climatol..

[2] Susanto RD, Wei Z (2016). Oceanography surrounding Krakatau Volcano in the Sunda Strait, Indonesia. Oceanography.

[3] Bryden HL, Imawaki S, Siedler G, Church J, Gould J (2001). Ocean transport of heat. Ocean Circulation and Climate.

[4] Sprintall J, Gordon AL, Koch-Larrouy A, Lee T, Potemra JT, Pujiana K, Wijffels S (2014). The Indonesian Seas and their role in the coupled ocean-climate system. Nat. Geosci..

[5] Lee T, Fukumori I, Menemenlis D, Xing Z, Fu LL (2002). Effects of the Indonesian throughflow on the Pacific and Indian Ocean. J. Phys. Oceanogr..

[6] Deckker P (2016). The Indo-Pacific warm pool: Critical to world oceanography and world climate. Geosci. Lett..

[7] Saji NH, Goswami BN, Vinayachandran PN, Yamagata T (1999). A dipole mode in the tropical Indian Ocean. Nature.

[8] Webster PJ, Moore AM, Loschnigg JP, Leben RR (1999). Coupled oceanic-atmospheric dynamics in the Indian Ocean during 1997–98. Nature.

[9] Ashok K, Guan Z, Yamagata Y (2001). Impact of the Indian Ocean dipole on the decadal relationship between Indian monsoon rainfall and ENSO. Geo. Res. Let..

[10] Barsugli JJ, Sardeshmukh DD (2002). Global atmospheric sensitivity to tropical SST anomalies throughout the Indo-Pacific Basin. J. Clim..

[11] Halkides D, Lee T, Kida S (2011). Mechanisms controlling the seasonal mixed-layer temperature and salinity of the Indonesian Seas. Ocean. Dyn..

[12] McBride J, Haylock M, Nicholls N (2003). Relationships between the maritime continent heat source and the El Niño–southern oscillation phenomenon. J. Clim..

[13] Miller M, Beljaars A, Palmer T (1992). The sensitivity of the ECMWF model to the parameterization of evaporation from the tropical oceans. J. Clim..

[14] Yamanaka MD (2016). Physical climatology of Indonesian maritime continent: An outline to comprehend observational studies. Atmos. Res..

[15] Matsumoto, J., Oki, T., Yamanaka, M.D., Hayashi T. & Asanuma, J. 10 years of MAHASRI: accomplishments and the international science conference wrap-up. *Gewex News* **26**, 10–15. http://www.gewex.org/gewex-content/files_mf/1480533350Nov2016_final_opt (2016).

[16] Matsumoto, J. *et al.* An overview of the Asian Monsoon Years 2007–2012 (AMY) and multi-scale interactions in the extreme rainfall events over the Indonesian maritime continent. in *The Global Monsoon System: Research and Forecast* (Chang, C.P. et al. Eds.). 3rd Ed. *World Scientific Series on Asia-Pacific Weather and Climate*. Vol. 9. 365–386. (World Scientific Publication Company, 2017).

[17] Yamanaka MD (2018). Maritime continent coastlines controlling Earth’s climate. Prog. Earth Planet Sci.

[18] Hamada J (2012). Interannual rainfall variability over northwestern Java and its relation to the Indian Ocean dipole and El Niño southern oscillation events. SOLA.

[19] Yamanaka MD (2020). Global and Indonesian climate in 2019. Newsl. Trop. Peatland Soc. Project (Research Institute for Humanity and Nature).

[20] Rafat A (2021). Non-growing season carbon emissions in a northern peatland are projected to increase under global warming. Commun. Earth Environ..

[21] Loisel J (2021). Expert assessment of future vulnerability of the global peatland carbon sink. Nat. Clim. Chang..

[22] Günther A (2020). Prompt rewetting of drained peatlands reduces climate warming despite methane emissions. Nat. Commun..

[23] Helbig M (2020). Increasing contribution of peatlands to boreal evapotranspiration in a warming climate. Nat. Clim. Change.

[24] Leifeld J, Wüst-Galley J, Page S (2019). Intact and managed peatland soils as a source and sink of GHGs from 1850 to 2100. Nat. Clim. Change.

[25] Huang Y, Ciais P, Luo Y, Zhu D, Wan Y (2021). Tradeoff of CO_2_ and CH_4_ emissions from global peatlands under water-table draw down. Nat. Clim. Change.

[26] Hoyt AM, Chaussard E, Seppalainen SS, Harvey CF (2020). Widespread subsidence and carbon emissions across Southeast Asian peatlands. Nat. Geosci..

[27] Cooper HV (2020). Greenhouse gas emissions resulting from conversion of peat swamp forest to oil palm plantation. Nat. Commun..

[28] Deshmukh CS (2021). Conservations lows down emission increase from a tropical peatland in Indonesia. Nat. Geosci..

[29] Osaki, M. &Tsuji, N. (Eds.). *Tropical Peatland Ecosystems*. (Springer, 2016).

[30] Hirano T (2012). Effects of disturbances on the carbon balance of tropical peat swamp forest. Glob. Change Biol..

[31] Mezbahuddin M, Grant RF, Hirano T (2014). Modeling effect of seasonal variation in water table depth on net ecosystem CO_2_ exchange of a tropical peatland. Biogeosciences.

[32] Taufik M (2017). Amplification of wildfire area burn the hydrological drought in the humid tropics. Nat. Clim. Change.

[33] Yulianti N, Hayasaka H (2013). Recent active fires under El Niño conditions in Kalimantan, Indonesia. Am. J. Plant Sci..

[34] Putra EI, Hayasaka H (2011). The effect of the precipitation pattern of the dry season on peat fire occurrence in the Mega Rice Project area, Central Kalimantan, Indonesia. Tropic.

[35] Shigenaga, Y. *et al.* Field data transmission system by universal mobile telecommunication network in (Osaki, M. & Tsuji, N. eds). *Tropical Peatland Ecosystems*. (Springer, 2016).

[36] Susanto RD, Gordon AL, Zheng Q (2001). Upwelling along the coasts of Java and Sumatra and its relation to ENSO. Geophys. Res. Lett..

[37] Santoso A, McPhaden MJ, Cai W (2017). The defining characteristics of ENSO extremes and the strong 2015/2016 El Niño. Rev. Geophys..

[38] Wirasatriya A (2020). Ekman dynamics variability along the southern coast of Java revealed by satellite data. Int. J. Remote Sens..

[39] Mandal S, Susanto RD, Ramakrishnan B (2022). Dynamical factors modulating surface Chlorophyll-*a* variability along South Java Coast. Remote Sens..

[40] Hameed SJ, Jin D, Thilakan V (2018). A model for super El Niños. Nat. Commun..

[41] Horii T, Hase H, Ueki I, Masumoto Y (2008). Oceanic precondition and evolution of the 2006 Indian Ocean dipole. Geophys. Res. Lett.

[42] Santoso A (2018). Dynamics and predictability of the El Niño-Southern Oscillation: An Australian perspective on progress and challenges. Bull. Am. Meteorol. Soc..

[43] Cai W, Wang G, Santoso A, Lin X, Wu L (2017). Definition of extreme El-Niño and its impact on projected increase in extreme El Niño frequency. Geophys. Res. Lett..

[44] Yoneyama K, Zhang C (2020). Years of the maritime continent. Geophys. Res. Lett..

[45] Ogino SY, Yamanaka MD, Mori S, Matsumoto J (2017). Tropical coastal dehydrator in global atmospheric water circulation. Geophys. Res. Lett..

[46] Steffen W (2015). Guiding human development on a changing planet. Science.

